# Borderline Personality Pathology in an At Risk Mental State Sample

**DOI:** 10.3389/fpsyt.2019.00838

**Published:** 2019-11-14

**Authors:** Tobias Paust, Anastasia Theodoridou, Mario Müller, Christine Wyss, Caitriona Obermann, Wulf Rössler, Karsten Heekeren

**Affiliations:** ^1^The Zurich Program for Sustainable Development of Mental Health Services (ZInEP), University of Zurich, Zurich, Switzerland; ^2^Department of Psychiatry, Psychotherapy and Psychosomatics, University of Zurich, Zurich, Switzerland; ^3^Laboratory of Neuroscience (LIM 27), Institute of Psychiatry, University of São Paulo, São Paulo, Brazil; ^4^Department of Psychiatry and Psychotherapy, Charité University Medicine, Berlin, Germany; ^5^Department of Psychiatry and Psychotherapy I, LVR-Hospital Cologne, Cologne, Germany

**Keywords:** borderline personality disorder, psychosis, at-risk, transition, basic symptoms, positive symptoms

## Abstract

**Introduction:** Transient psychotic symptoms in patients with borderline personality disorder seem to be similar to those in patients with psychotic disorders. Especially in the field of early detection of psychosis, this might lead to individuals with borderline personality disorder being wrongly classified as subjects at risk for developing a manifest psychosis. The aim of the present study was to investigate the occurrence of borderline symptoms in a sample of subjects at risk for psychosis as well as possible effects on the transition rate.

**Methods:** Seventy help-seeking individuals of an early psychosis recognition center were additionally examined for borderline symptoms by the borderline symptom checklist.

**Results:** We found a significant correlation between borderline symptomatology and positive symptoms assessed by the structured interview for prodromal symptoms. There were no associations between basic symptoms for psychosis and borderline symptoms. In addition, there was no influence of borderline symptomatology on the rate of transition into a manifest schizophrenic disease.

**Summary:** In conclusion, borderline personality disorder should not be an exclusion criterion for the screening for psychosis or for an early intervention treatment. On the other hand, not every patient with borderline personality disorder, (especially those not suffering from hallucinations, unusual thought content, or persecutory ideas) should automatically be screened for the risk of developing a psychotic disorder.

## Introduction

Early views of borderline personality disorder (BPD) were based on the idea that patients with this pathology were “on the border” of psychosis (1). Historically, Stern used the term borderline to describe patients who showed neurotic and psychotic symptoms simultaneously (2). The current definition of BPD contains significant impairments in personality functioning and impairments in interpersonal functioning like empathy and intimacy. Pathological personality traits usually occur in the following domains: *Negative Affectivity* - characterized by emotional liability, anxiousness, separation insecurity, and depressivity; *Disinhibition* - characterized by impulsivity and risk taking;

*Antagonism* - characterized by hostility.

According to the *Diagnostic and Statistical Manual of Mental Disorders–Fifth Edition* (DSM-5), psychotic symptoms such as auditory verbal hallucinations (AVH) and other “positive” symptoms of psychosis may be present in patients with BPD, but occur “only for brief periods in situations of distress” (3).

Recent studies suggest that, in patients with BPD, some hallucinations, like hearing voices, seem to be similar to those experienced by patients with psychotic disorders (4). Since the presence of attenuated psychotic symptoms is not unusual in BPD, it can be difficult to make the right diagnosis. The separation between the two diagnoses is even more difficult as there is some evidence that 10% of patients initially diagnosed with BPD actually do transition to a psychotic diagnosis (5).

Lifetime prevalence of schizophrenia is around 0.7% (6) and lifetime prevalence of BPD is mentioned to be four times higher at around 3% (7). While schizophrenia is often associated with a variety of concurrent psychiatric symptoms, initially, little attention was paid to the prevalence of comorbidity in patients at risk for schizophrenic psychosis (8).

At risk mental state (ARMS) is a term which is used by health professionals to describe adolescents and adults who are experiencing perceptual changes that may be early, low level, signs of psychosis (9). The at risk mental state term is actually used more commonly than the prodromal term because the prodromal term implies the inevitable onset of the illness rather than the fact that the course of the illness is variable (10). This means that “prodrome” is necessarily a retrospective concept (11).

Yung and colleagues (12) developed clinical criteria like the ultra-high-risk (UHR) criteria to identify individuals at risk for psychosis. Specific assessment tools like Comprehensive Assessment of at Risk Mental State (CAARMS) (13) and Structured Interview for Prodromal Symptoms (SIPS) provide scores of symptom domains and they defined three states that lead to the three different UHR criteria (14):

1. transient psychotic symptoms (brief limited psychotic symptoms, BLIPS)
2. attenuated psychotic symptoms (APS)
3. genetic risk combined with a functional decline

The presence of one of this three criteria is sufficient to fulfill the UHR state.

Another approach based on self-experienced subtle subclinical symptoms, the so called basic symptoms, was first described by Gerd Huber (15, 16). Basic symptoms are mainly subjectively experienced disturbances in motor action, perception, affect, thinking, speech, stress tolerance, and central vegetative functions (17). These concepts were mainly developed to detect the risk of psychosis as early as possible, ideally before functional impairments appear (18). They are thought to be the most immediate symptomatic expression of the neurobiological correlate of the psychotic illness (19). Basic symptoms are assessed with a structured manual (e.g., Schizophrenia Proneness Instrument, SPI-A) to enable a reliable detection and scoring of this symptoms (20).

Meanwhile, criteria from UHR are combined with the basic symptoms to increase the accuracy of prediction. The EPA guidance on early detection of clinical high-risk states currently recommends to use attenuated psychotic symptoms or COGDIS or BLIPS criteria alternatively (18).

Historically, our understanding of the early course of schizophrenia primarily has been limited to a retrospective assessment after the onset of the psychotic phase (8). During the last 20 years, research has focused on detecting psychosis in the prodromal stage by prospective studies. The average duration of the prodromal phase is 5 years (21), which should give the clinician enough time to begin with suitable interventions. Therefore, a number of studies focus on this prodromal phase in order to develop diagnostic and intervention strategies, for example:

PACE: Personal Assessment and Crisis Evaluation (22)
PRIME: Prevention through Risk Identification and Education (23)
EPOS: European Prediction of Psychosis Study (24)
NAPLS: North American Prodromal Longitudinal Study (25)
ZInEP: Zürcher Impulsprogramm zur nachhaltigen Entwicklung in der Psychiatrie (26)
PRONIA: Personalized Prognostic Tools for Early Psychosis Management (27)

On average 20% of the individuals who meet the basic symptom criteria make the transition to psychosis in the first year after presentation (28). Individuals who fit the UHR-Criteria were initially found to have rates of transition to psychosis of around 40% in the first 12 months after presentation (29, 30). Fusar-Poli et al. (31) made a systematic review of at risk studies and found an average transition rate of 36% after three years. However, this transition rate has dropped to rates as low as 15% in recent studies. One explanation of the declined transition rate could lie in the increased awareness of attenuated psychotic symptoms, which consequently led to an earlier and more effective intervention to prevent the progression to psychosis (32). It has also been argued that the decline of transition rates could be explained by a treatment effect, for example with antipsychotics (33).

As the concept of at-risk mental states has gained extensive community awareness over the years, the so-called dilution effect has also been postulated. An increased attentiveness to at-risk mental state may have been associated with less selective referral patterns, in turn leading to a possible dilution of the pool of young people who are screened using the UHR criteria (33). This could explain the higher proportions of false-positive predictions in the more recent studies, which consequently may lead to stigma and unnecessary treatment (10).

A further problem of early detection of psychosis results from the lack of clear boundary between subthreshold and manifest psychotic symptoms. Meanwhile, there is much evidence for a continuum of psychosis from subclinical psychotic symptoms in general population (and conscripts) without indication for treatment up to manifest schizophrenia (34, 35). Moreover, persons with a subclinical disorder show rising and declining symptom states in the long-term course assessed with the Symptom-Checklist-90-R (36). Thus, depending on their symptom fluctuations, some persons might meet criteria for either at risk for psychosis or manifest schizophrenia over time. That means these people show a dynamic course going back and forth across the diagnostic threshold of psychosis (37).

Another reason for a false-positive prediction could lie in the comorbidity in ultra-high-risk samples. Fusar-Poli et al. (38) showed in their study that comorbid depression and anxiety diagnoses had no effect on the risk of transition to psychosis. On the other hand, there is a prevalence of more than 40% for any personality disorder in several UHR samples (17). Mainly, the borderline personality disorder is common in UHR-Patients (5, 7). The association between BPD and the ultra-high-risk is unclear (39). Personality dimensions can be seen as vulnerability markers, which may increase the risk for psychosis in normal subjects (40). In a case control study from Thompson et al. (5), the presence of borderline personality feature was not associated with a reduced risk of transition to psychosis but they suggest that the types of attenuated psychotic symptoms might be different in UHR individuals with BPD compared with UHR-individuals without BPD features. What does this mean for the clinician? Is it important to screen patients with BPD for psychosis as well? We hypothesized that might be some individuals with borderline personality disorder which are falsely staged to the UHR state and that these individuals will not make the transition to psychosis. Additionally, we expected that there would be a significant correlation between borderline personality symptomatology and positive symptoms that predict the UHR state in our ZInEP sample. Furthermore, we wanted to investigate whether the types of attenuated psychotic symptoms are different in UHR individuals with BPS compared with UHR-individuals without BPS. Finally, we tried to figure out whether there is a correlation between borderline personality symptomatology and basic symptoms.

## Materials and Methods

### Sample

The sample contains 70 help-seeking individuals of the (ZInEP) early psychosis recognition center (26). These individuals were referred either by a general practitioner or psychiatrist to evaluate the risk of psychosis or they came by their own means after noticing certain symptoms and being afraid of developing a psychosis. All subjects were examined for both the presence of the UHR-state and the basic symptom criteria. The sample was then divided into individuals meeting the ultra-high-risk (UHR) criteria, the basic symptom (BS) criteria, or not meeting any of both criteria (for details and demographic data, see *Results* and **Table 1**). The borderline symptom checklist was assessed for all subjects in all three groups. Exclusion criteria were any substance addiction disorder, presence of a current, or past manifest psychotic disorder, any medical condition known to affect the brain or an estimated verbal IQ < 80. The study was approved by the local ethic committee and written informed consent was obtained before study enrolment.

**Table 1:** Demographic and clinical characteristics.

	CN	BS	UHR	Test statistics
n	10	34	26	
Gender (F:M)	6:4	19:15	14:12	X2 = 0.112, p = 0,946
Age	22.2 ( ± 4.89)	23.59 ( ± 5.13)	19.88+-5.61	F = 3.43, p = 0,038
SIPS positive	3.4 (±-2.5)	4.15+-2.74	10.96+-3.55	F = 43.23, p < 0.001
SIPS negative	9.4+-4.6	9.76+-5.86	14.15+-6.82	F = 4.4, p = 0.016
SIPS global	5.3+-3.88	6.91+-3.39	9.39+-3.6	F = 6.09, p = 0.004
CPZe (Medication)	5.7+-18.02	9.12+-29.34	50.81+-91.87	F = 4.08, p = 0.021
BSL 23	16.1+-11.25	20.5+-15.14	36.42+-17.8	F = 9.73, p < 0.001

CN, criterion negative; BS, basic symptom; CPZe, antipsychotic medication status in chlorpromazine-equivalent dosage; UHR, ultra high risk (41).

### Assessment

Basic symptoms were assessed by the schizophrenia proneness instrument (SPI-A). The SPI-A is a further development of the BSABS (Bonner Skala zur Beurteilung von Basissymptomen) based on a sample of retrospective prodromal patients. The scale consists of 40 items and six dimensions: affective-dynamic disturbances, cognitive-attentional impediments, cognitive disturbances, disturbances in experiencing the self and surroundings, body perception disturbances, and perception disturbances (20).

The **basic symptom** (BS) risk state for psychosis was defined by two basic symptom criteria:

1. **COPER-**criterion: Presence of at least any one of the cognitive-perceptive basic symptoms with a SPI-A score of at least 3: thought interference, thought perseveration, thought pressure, thought blockages, disturbance of receptive speech, decreased ability to discriminate between ideas and perceptions, unstable ideas of reference, derealisation, visual perception disturbances, and acoustic perception disturbances.
2. **COGDIS-**criterion: Presence of at least any two of the following cognitive disturbances with a SPI-A score of at least 3: Inability to divide attention, thought interferences, thought pressure, thought blockages, disturbance of receptive speech, disturbances of expressive speech, unstable ideas of reference, disturbances of abstract thinking, and captivation of attention by details of the visual field.

COPER and COGDIS symptoms need to be present over the last 3 months and, for COPER, in addition, the first occurrence has to be more than 12 months ago.

The **UHR-state** was assessed by the structured interview for prodromal symptoms (SIPS) (14):

1. **APS**: At least one attenuated psychotic symptom with a score between 3 and 5 in the SIPS score. The symptoms must have begun during the last 12 months or need to score at least one point higher than one year before. They need to occur with a mean frequency of at least once a week during the last months.
2. **BLIPS** (Brief limited psychotic symptoms): At least one symptom with the score 6 in the SIPS score. The symptoms must have started during the last 12 months and must occur at least a few minutes a day with a frequency of at least once a month. They need to regress without any intervention.
3. **State-trait criteria**: A first-degree relative with a history of psychosis or a schizotypal personality disorder plus a reduction of 30% in the General Assessment of Function Scale lead to the state-plus-trait criteria of UHR.

The **borderline personality** dimensions were assessed by the borderline symptom checklist (BSL-23). This is a short version from the BSL-95. The BSL items are based on criteria of the DSM 4, the revised version of the Diagnostic Interview for BPD, and the opinion of clinical experts and BPD patients. The questionnaire uses a Likert-type rating format: 0 = not at all; 1 = a little; 2 = rather; 3 = much; and 4 = very strong. Data from an evaluation study suggest that the BSL-23 has good to excellent psychometric properties similar to the BSL-95, its ability to discriminate BPD patients from other psychiatric patients is high (42). The BSL is strongly associated with the presence of DSM 4 BPD symptoms assessed by a valid semistructured interview, although a cut-off score is missing (43). We defined a significant borderline symptomatology in scores at least one standard deviation higher than the mean score).

The medication of the subjects was assessed and chlorpromazine-equivalent dosages (CPZ) were calculated using the criteria of Andreasen et al. (41).

All subjects were followed up over three years as part of the ZInEP early recognition study (26, 44) to detect transitions in a manifest psychotic disorder.

### Data Analyses

Statistical analyses were performed by Statistical Package for Social Science (SPSS) for windows version 24 (SPSS Inc. Chicago, IL, USA). Demographic and clinical characteristics between groups were compared by using Chi-squared and Fishers exact test for categorical variables or one-way ANOVA for continuous variables. To examine the influence of age and antipsychotic medication, both age and CPZ were included as covariate in the ANOVA. Kendall tau b correlations were used to calculate the association between BSL-23 and SIPS as well as SPI-A. Due to the strong overlap in the range of positive symptoms, we calculated correlations between the positives scores of the SIPS and the BSL.

## Results

**Figure 1:**
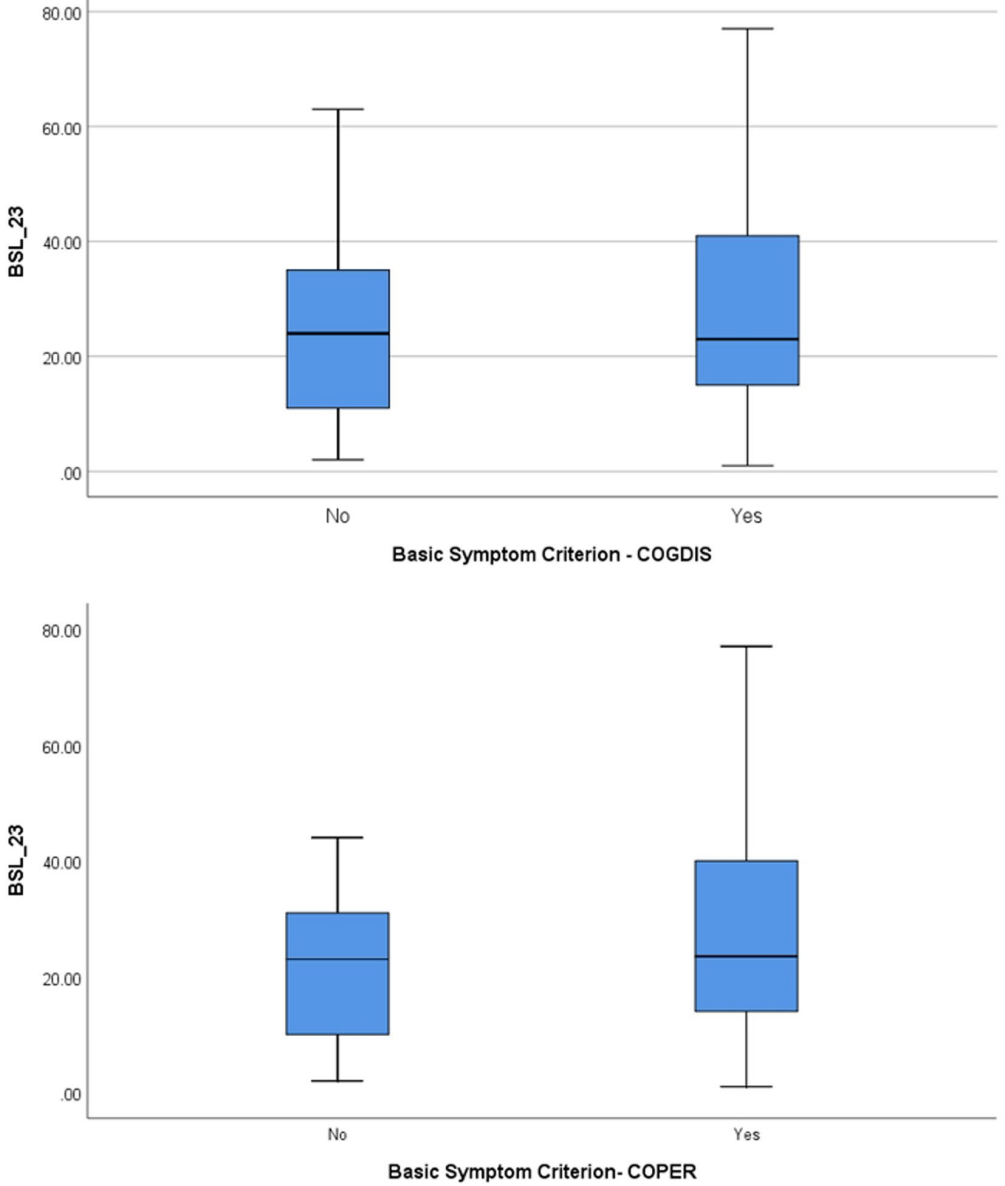
Borderline symptom list (BSL) score separated according to the presence of basic symptom criteria COPER and COGDIS.

Twenty-six (37%) individuals of the sample (N = 70) met Criteria for UHR (of these: 20 met only APS, three APS and BLIPS, two only BLIPS, and one met only genetic risk and functional decline). Thirty-four (48.5%) individuals exclusively (without UHR) met the basic symptom criteria (of these: 13 met only COPER and 21 COPER and COGDIS), while 10 (14.2%) met neither of those risk criteria (**Table 1**). Bonferroni post-hoc comparisons revealed that the UHR subjects had significant higher BSL mean scores than BS and CN (criterion negative) subjects. Within the BS group, there was no difference in BSL scores between COPER and COGDIS (see **Figure 1**).

**Figure 2:**
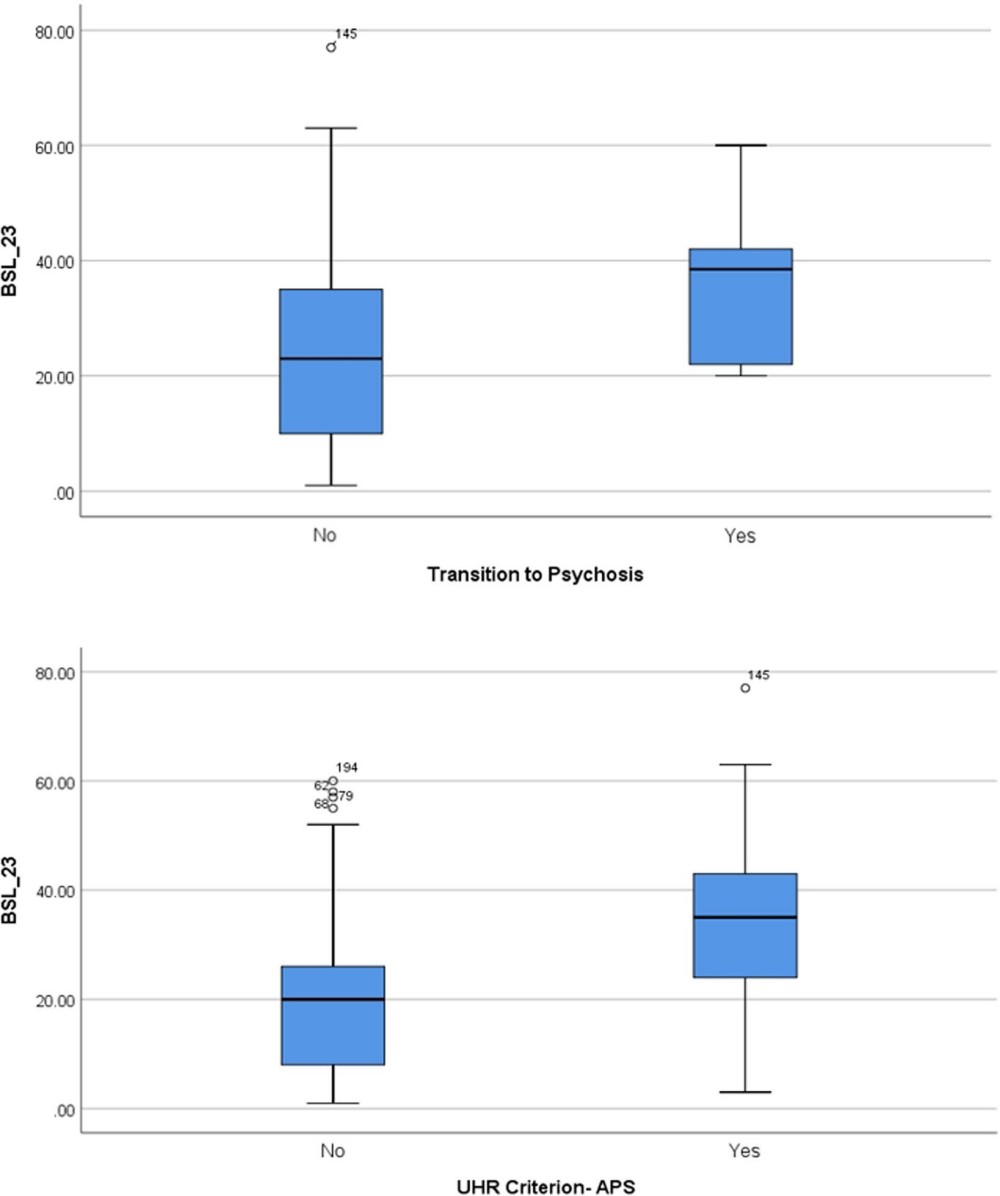
Borderline symptom list (BSL) score separated according transition into manifest psychosis and according to the presence of the attenuated positive symptom (APS) criterion.

No significant difference in BSL scores was found between subjects with transition into a manifest schizophrenic disorder (according to ICD-10) and subjects at risk for psychosis without transition. In addition, there was no significant difference with respect of attenuated psychotic symptoms (see **Figure 2**). There were also no statistical significant differences in BSL-scores in gender and age.

Since there was a significant difference in age and medication between the three groups, we performed a second ANOVA with age and CPZ (chlorpromazine-equivalent) (41) as a covariate. The second ANOVA also revealed a significant difference of the BSL score between the three groups (F = 6.78; p = 0.002). Post hoc: UHR versus BS p = 0.007 and UHR versus CN = 0.008). However, the SIPS negative score did no longer differ significantly between the three groups when age and CPZ used as a covariate.

In the correlation analysis of positive symptoms, we found a significant correlation between the BSL score and the following three SIPS positive items: “unusual thought content,” “suspiciousness/persecutory ideas,” and “perceptual abnormalities/hallucinations.” The correlation between the BSL score and the other two SIPS Items “grandiosity” and “disorganized communication” were not statistically significant (**Table 2**).

**Table 2:** Correlation (Kendall tau b) between borderline symptom checklist (BSL 23) and Structured Interview for Prodromal Symptoms (SIPS) positive items.

	SIPS P1 Unusual Thought content	SIPS P2 Suspiciousness/persecutory ideas	SIPS P3 Grandiosity	SIPS P4 Hallucinations	SIPS P5 Disorganized communication
BSL 23	r = 0.202 **p = 0.024**	r = 0.201 **p = 0.026**	r = 0.128 p = 0.181	r = 0.228 **p = 0.012**	r = 0.088 p = 0.346

Significant p-values in bold.

Furthermore, there was no significant correlation between basic symptoms which define the high risk state and the BSL score (**Supplement Table S1**).

In our sample of 70 patients, 60 were rated as at risk mental state. Six of these individuals transitioned to psychosis leading to a transition rate of 10%. As a cutoff score of the BSL does not exist, we defined a significant borderline symptomatology in the range of one standard deviation above the mean score. In our sample, this was a score higher than 42 on the BSL. Based on this definition, 11 subjects in our sample had a pronounced and relevant borderline symptomatology. Only one of these 11 subjects made a transition to manifest schizophrenia; thus, the transition rate is 9.1%. The group with low borderline symptomatology (BSL-scores below 42) contained 49 individuals and 5 transitions, leading to a transition rate of 10.2%. Hence, the transition rates are almost equal.

## Discussion

Transient hallucinations are a common symptom in BPD as well as a common attenuated positive symptom, which is part of the UHR state. Therefore, in patients with a BDP, it is difficult to determinate whether transient hallucinations are a symptom of the BPD alone or if they in fact pose as a risk factor for a transition to psychosis. As we see in the significant correlation between BSL-scores and some important “prodromal” positive symptoms of schizophrenia (like unusual thought content, suspiciousness and hallucinations) in the SIPS score, it is difficult to determine whether the symptoms are part of a at risk mental state or the borderline personality. Thus, longitudinal studies with several follow up examinations are needed. If the score of the hallucinations turns out to stay equal, the hallucinations may possibly be part of the borderline personality symptomatology alone. If on the other hand the score increases, this may predict a transition to psychosis. Patients with borderline symptoms should also be screened and treated for the risk of developing a psychotic disorder if they present attenuated psychotic symptoms or basic symptoms that meet an ultra high risk or basic symptom risk state. As we found no statistical significant correlation between the BSL-score and the basic symptom risk states COPER and COGDIS, any patient with BPD who presents basic symptoms meeting the high-risk state should be observed and treated concerning the risk of making a transition to psychosis.

The fact that we found almost equal transition rates in the group with low borderline personality symptomatology (10.2%) and high borderline personality symptomatology (9.1%) is a contradiction to our suggestion, that UHR individuals with high BSL-scores (more than one standard deviation to the mean score) show lower rates of transition to psychosis. Concerning these results, we have to keep the limitations of the study in mind. First of all, it is a small sample (70 individuals) with only six transitions, in which we only have one transition in the group with high borderline personality symptomatology. Further studies with a larger number of cases are needed in order to clarify the correlation between borderline symptomatology and risk for psychosis. In addition, future investigations should investigate the comorbidity of psychotic symptoms in other cluster A and B personality disorders too. Another limitation is the self- rating Instrument BSL 23 with missing cut off scores, so that we could not define the state of a significant borderline symptomatology exactly. All in all, these results show that it is very important to focus on individuals with symptoms of BPD as well when researching a UHR cohort.

As we suggested, some of the APS items like unusual thought content, suspiciousness/persecutory ideas, and perceptual abnormalities/hallucinations that lead to the UHR state have a strong correlation to the BSL-scores. These are in general the psychotic symptoms which are seen clinically often in borderline personality disorder. Psychotic symptoms in patients with borderline personality disorder usually occur for brief periods of time in stressful situations. Severe difficulties in interpersonal functioning can induce the development of undue suspiciousness, ideas of reference, and other symptoms of nondelusinal paranoia (45). The UHR state is characterized by brief psychotic or attenuated psychotic symptoms (BLIPS and APS) which might be induced by stress. Moreover, hallucinations, especially auditory verbal hallucinations, are a common symptom in borderline personality disorder (46). The two SIPS items that did not correlate with the BSL (grandiosity and disorganized communication) are usually not seen in BPD.

Furthermore, we should keep the existing comorbidity of BPD and schizophrenia in mind. There are only a few studies that have examined BPD in individuals with schizophrenia and the results differ considerably ranging between 3% and 25% (47). BPD seems to occur more often in UHR and first episode patients. This means that up to a quarter of first episode patients could suffer from this comorbidity (48). In the study from Bahorik and Eack (47), patients with schizophrenia and BPD improved less in overall psychiatric symptomatology, had poorer global functioning, and were rehospitalized at significantly higher rates than patients with schizophrenia alone. As this comorbidity often leads to severe illness and difficulties in compliance, it is necessary to recognize this risk as early as possible in order to provide an adequate treatment.

We conclude that the diagnosis of borderline personality disorder should not be an exclusion criterion for the screening for psychosis or for an early intervention treatment. In particular, the basic symptoms appear to be completely independent of comorbid borderline symptoms. On the other hand, not every patient with BPD (especially those not suffering from hallucinations, unusual thought content or persecutory ideas) should automatically be screened for the risk of developing a psychotic disorder.

## Data Availability Statement

The datasets generated for this study are available on request to the corresponding author.

## Ethics Statement

The study was approved by the regional ethics committee of the canton of Zurich (KEK-ZH-Nr. E-63/2009) and written informed consent was obtained before study enrolment.

## Author Contributions

TP, AT, WR, and KH designed the study and wrote the protocol. TP and CW collected the data. TP, MM, and KH analyzed the data. TP drafted the manuscript. TP, AT, MM, CW, CO, WR, and KH discussed the results and reviewed the manuscript, making critical revisions. All authors contributed to and have approved the final manuscript.

## Funding

The Zurich Program for Sustainable Development of Mental Health Services (ZInEP) was supported by a private donation. The donor had no further role in the experimental design, collection, analysis, interpretation of data, writing, and submitting this paper for publication.

## Conflict of Interest

The authors declare that the research was conducted in the absence of any commercial or financial relationships that could be construed as a potential conflict of interest.
