# Supplementary material for: Borderline Personality Pathology in an At Risk Mental State Sample
Source: Front Psychiatry. 2019 Nov 14;10:838. doi: 10.3389/fpsyt.2019.00838 (PMC6874151; doi:10.3389/fpsyt.2019.00838)
Supplement: Supplementary file 1 [file Table_1.docx]

Supplement table s1:

Correlation (Kendall tau b) between BSL 23 and basic symptoms:

| **basic symptom:** | **BLS-23** |
| --- | --- |
| inability to divide attention | r=0.153, p=0.119 |
| disturbance of expressive speech | r=0.054, p=0.576 |
| disturbances of abstract thinking | r=-0.097, p=0.318 |
| captivation of attention by details of the visual field | r=-0.053, p=0.579 |
| thought interference | r=0,098, p=0.292 |
| thought pressure | r=0.089, p=0.333 |
| thought blockages | r=0.157, p=0.086 |
| disturbance of receptive speech | r=-0.050, p=0.606 |
| unstable ideas of reference | r=-0.010, p=0.918 |
| thought perseveration | r=0.120, p=0.216 |
| derealization | r=-0.038, p=0.681 |
| visual perception disturbances | r=0.094, p=0.336 |
| acoustic perception disturbances | r=0.042, p=0.667 |
| decreased ability to discriminate between ideas and perception, fantasy and true memories | r=0.126, p=0.186 |
